# Perspective of Human Condensins Involved in Colorectal Cancer

**DOI:** 10.3389/fphar.2021.664982

**Published:** 2021-09-07

**Authors:** Hongzhen Wang, Yao Chen, Dawei Yang, Liang Ma

**Affiliations:** ^1^ School of Life Sciences, Jilin Normal University, Siping, China; ^2^ The Department of General Surgery, The Central People’s Hospital of Siping City, Siping, China

**Keywords:** condensin, human condensin, structure of chromosomes protein, abnormal expression, colorecal cancer

## Abstract

Although many important roles are played by human condesins in condensation and segregation of mitotic chromosomes, what roles of human condensins play in colorectal cancer are still unclear at present. Recently, abnormal expressions of all eight subunits of human condensins have been found in colorectal cancer and they are expected to become potential biomarkers and therapeutic targets for colorectal cancer in the future. However, there are still no reviews on the significance of abnormal expression of human condensin subunits and colorectal cancer until now. Based on a brief introduction to the discovery and composition of human condensins, the review summarized all abnormally expressed human subunits found in colorectal cancer based on publicly published papers. Moreover, Perspective of application on abnormally expressed human subunits in colorectal cancer is further reviewed.

## Introduction

Nowadays colorectal cancer (CRC) is the second leading cause of cancer mortality and the third most commonly diagnosed cancer worldwide (Bray et al., 2018). With the rapid development of the economy, life expectancy correlated positively with the incidence and mortality of CRC both in men and women in China (Gu et al., 2018). Although there has been a clear treatment method for the early colorectal cancer with general surgery and adjuvant chemotherapy, many colorectal cancer patients are in the late stage when they were firstly identified because of the lack of effective biomarkers in early screening of colorectal cancer and they are hard to cure. Therefore, it is of great significance to strengthen the study of molecular mechanism of colorectal cancer and develop effective biomarkers and therapeutic targets for early screening and diagnosis of colorectal cancer. The identification of abnormal expression proteins of colon cancer is pivotal for early detection of colorectal cancer and effective development of biomarkers and therapeutic targets.

Recently, all eight human condensin subunits have been found abnormally expressed in colorectal cancer and they are expected to become potential biomarkers and therapeutic targets for colorectal cancer in the future (Dávalos et al., 2012; Shiheido et al., 2012; Tokunaga et al., 2013; Feng et al., 2014; Je et al., 2014; Jinushi et al., 2014; Yin et al., 2017; Baergen et al., 2019; Montero et al., 2020; Yeh et al., 2020). Therefore, based on the brief introduction of the discovery and composition of human condensins, this paper reviews the research progress and application perspective of all eight human condensin subunits in colorectal cancer to offer beneficial reference for related research.

### Discovery and Composition of Human Condensins

In 2001, human condensin I was firstly purified based on the study of human ortholog of frog condensin from HeLa nuclear extracts (Hirano and Mitchison, 1994; Schmiesing et al., 2000; Kimura et al., 2001). In 2003, Ono et al. (2003) discovered another condensin complex in HeLa nuclear extracts and the two kinds of condesins in human cells are termed as condensin I and II respectively (Kimura et al., 2001).

Both of human condensins are composed of five evolutionally conserved subunits. SMC2 (also named as human chromosome associate polypeptide E, hCAP-E) and SMC4(hCAP-C)are formed heterodimer and shared by the two kinds of human condesins (Schmiesing et al., 1998; Kimura et al., 2001; Ono et al., 2003). The other three non-SMC subunits are hCAP-D2 (NCAPD2), hCAP-H (NCAPH) and hCAP-G (NCAPG) in human condensin I and hCAP-D3 (NCAPD3), hCAP-H2 (NCAPH2) and hCAP-G2 (NCAPG2) in human condensin II (Kimura et al., 2001; Ono et al., 2003). Although the two kinds of human condensins have similar composition and alphabetic structure, they play different roles in the chromosome dynamics during the cell cycle (Hirota et al., 2004; Ono et al., 2004; Onn et al., 2007).

### All Eight Subunits of Human Condensins are Abnormally Expressed in Colorectal Cancer

All eight subunits of human condensins have been reported to be abnormally expressed in colorectal cancer until now, as shown in Table 1 (Dávalos et al., 2012; Shiheido et al., 2012; Tokunaga et al., 2013; Feng et al., 2014; Je et al., 2014; Jinushi et al., 2014; Yin et al., 2017; Baergen et al., 2019; Montero et al., 2020; Yeh et al., 2020; Zhang et al., 2021).

**TABLE 1:** All eight subunits of human condensins involved in colorectal cancer.

Human condensin subunits involved in colorectal cancer	Mutated gene/protein expression	Number of related literature and published year
hCAP-G2(NCAPG2)	unclear/expression of hCAP-G2 is affected by binding of Q15 or Q15 and MIP-2A	Shiheido et al., 2012
		Tokunaga et al., 2013
All eight human condensin subunits	gene copy number alterations include deletions, small-scale gains and/or large-scale amplifications/reduced expression	Baergen et al., 2019
SMC2(hCAP-E)	unclear/overexpression	Dávalos et al., 2012
	frameshift mutation/null	Je et al., 2014
	not mutated/reduced expression	Montero et al., 2020
	unclear/unclear	Yeh et al., 2020
SMC4(hCAP-C)	unclear/overexpression	Dávalos et al., 2012
	unclear/overexpression	Feng et al., 2014
	unclear/overexpression	Jinushi et al., 2014
	unclear/upregulated	Zhang et al., 2021
hCAP-H(NCAPH)	missense and frameshift mutation/overexpression	Yin et al., 2017

Firstly, expression of hCAP-G2, a non-SMC subunit of human codensin II, is affected by binding of Q15 (an anilinoquinazoline derivatives) and inhibiting proliferation of multiple cultured colorectal cancer cell lines (Shiheido et al., 2012). Furthermore, Q15 can also binds to MIP-2A (MBP-1 interacting protein-2A) and simultaneous targeting of hCAP-G2 and MIP-2A may be a promising strategy for the treatment of intractable colorectal tumors (Tokunaga et al., 2013).

Secondly, hypomorphic expression or no expression of SMC2 (hCAP-E) is implicated in cancer pathogenesis. Concretely, SMC2 gene is frameshift mutated because of mononucleotide repeats and this causes loss of its expression in colorectal cancer with high microsatellite instability (MSI-H) (Je et al., 2014). Likewise, reduced expression of all condensin genes may drive chromosome instability (CIN) and may contribute to colorectal cancer pathogenesis (Baergen et al., 2019). Consistent with this, inhibition of tumorsphere formation in colon cancer cell lines was observed recently when SMC2 was inhibited by intracellular delivery of specific anti-SMC2 antibodies (Ab-SMC2) alone or with anticancer drug by polymeric micelles (PM) (Montero et al., 2020). On the other hand, overexpression of SMC2 has also been found in both colorectal cancer cell lines and samples from CRC patients (Dávalos et al., 2012). The difference between overexpression and low expression of SMC2 may because that it is regulated by different signaling pathways. SMC2 promoter could be driven by WNT signaling and it cause SMC2 abnormal expression correlated with β-catenin levels in CRC cell lines and clinical samples (Dávalos et al., 2012). Similarly, it is reported that the receptor glutamate ionotropic receptor delta type subunit 2 (GRID2) binds ligand cerebellin 1 precursor (CBLN1) to transmit the signal to TF POU class 2 homeobox 1 (POU2F1) through SMC2 (Yeh et al., 2020). This signal upregulates the target gene hematopoietic prostaglandin D synthase (HPGDS) and inhibits the migration and proliferation of CRC cells (Tippin et al., 2012). These results mentioned above suggest that dysfunction of SMC2 is involved in pathogenesis of colorectal cancer.

Thirdly, SMC4 also shows dysregulations in colorectal cancer cells. For example, overexpression of SMC4 was found in both colorectal cancer cell lines and samples from CRC patients and downregulation of SMC4 plays a suppressive role in the proliferation of colorectal cancer cells and tumor growth (Dávalos et al., 2012; Feng et al., 2014). In addition, it is demonstrated that expression of SMC4 mRNA is downregulated by miR-124-5p (also known as miR-124*) and growth of human colon adenocarcinoma cell is inhibited. Therefore low expression levels of microRNA-124-5p and overexpression of SMC4 is correlated with poor prognosis in colorectal cancer (Jinushi et al., 2014). Consistent with this, recently Zhang et al. (2021) finally identified two molecular subtypes that had been named C1 and C2 based on the cell cycle-related genes in patients with colon cancer and SMC4 were identified to be one of fifty upregulated genes as markers for C2 subtypes.

Finally, NCAPH is overexpressed in colorectal cancer cell lines comparing with normal human colonic epithelial cells. Many NCAPH mutations include missense and frameshift are identified in colorectal cancer patients (Yin et al., 2017). Of note, contrast to SMC4, the patients with NCAPH overexpression in colon cancerous tissues had a significantly better prognosis and survival rate than those with low-expression of NCAPH in tumor tissues.

## Conclusion and Future Perspectives

Taken together, all eight human condensin genes have been found to be abnormally expressed in colorectal cancer until now. Although there are differences in different colorectal cancers with upregulation or downregulation of individual human condensin subunit, it just shows that colorectal cancer has complex signal transduction and genomic and transcriptional heterogeneity (Kyrochristos and Roukos, 2019).

At present, the identification of tumor specific targets or characteristics is of great significance for the treatment of cancer patients (Andre et al., 2014). The development of potential biomarkers to assist individualized therapy and drug discovery is becoming a new trend in the diagnosis and treatment of colorectal cancer (Zhai et al., 2017). The discovery of abnormal expression of human condensin subunits in colorectal cancer opens up a new way for the diagnosis and treatment of colorectal cancer. With the development of evidence-based colorectal cancer precision medicine, all eight condensin subunits could be potential biomarkers and therapeutic targets for colorectal cancer (Dávalos et al., 2012; Shiheido et al., 2012; Feng et al., 2014; Jinushi et al., 2014; Yin et al., 2017; Baergen et al., 2019; Yeh et al., 2020). However, the molecular mechanism of how they function in the process of tumorgenensis remains to be further explored in the future. There are three aspects worthy of attention about the future application research.

Firstly, the molecular mechanism of eight human condensin subunits involved in colorectal cancer needs further research. What roles each human condensin subunit plays in pathology of colorectal cancer are not very unclear. Whether the instability of chromosome is affected by the activity of the whole complex of human condensins or by some unknown signal transduction of each human condensin subunit is also unclear. Recently, it is reported that reduced condensin expression and function may be a significant, yet, underappreciated driver of colorectal cancer (Baergen et al., 2019). On the other hand, according to the idea that the human condensin subunits plays a role by affecting the function of the whole human condensin complex, Palou et al. (2018) proposed a cancer treatment hypothesis based on the overexpression of SMC4, a subunit of human condensin. SMC4 gene is often overexpressed in cancer cells, which exceeds the activity required for chromosome condensation in normal cells. Therefore, some inhibitors that inhibit the activity of condensin may kill cancer cells without damaging normal cells, and appropriately inhibiting the activity of ATPase of human condensin may selectively kill cancer cells without damaging normal cells.

Secondly, the microRNAs that regulate the expression of eight human condensin subunits in colorectal cancer need further research. MicroRNA can be developed as a biomarker for colorectal cancer (Ahmed, 2014; Qin and Liu, 2019; Farace et al., 2020). For example, mir-124-5p plays a role by regulating the expression of SMC4 target gene, which can be used as a biomarker in the diagnosis and treatment of colon cancer patients (Jinushi et al., 2014). The results suggest that microRNAs specifically target the other seven human condensin subunits may also be developed as potential biomarkers and therapeutic targets for colorectal cancer.

Finally, the interaction between the reported biomarkers of colorectal cancer and eight human condensin subunits in colorectal cancer need further research. Until now, multiple biomarkers for colorectal cancer have been found (Ye et al., 2016; Li et al., 2019; Erfani et al., 2020; Harvey et al., 2020; Huang et al., 2020; Lu et al., 2020). Nevertheless, it is unclear whether and/or how these biomarkers interact with the eight human condensin subunits in colorectal cancer.

Moreover, hCAP-D2(NCAPD2) and hCAP-D3(NCAPD3), non-SMC subunit of human condensin I or condensin II respectively, are overexpressed in patients with ulcerative colitis and the overexpression of these two proteins is regulated by IKK/NF-κB signal transduction pathway to promote the release of inflammatory cytokines (Yuan et al., 2019). Similarly, as mentioned above, SMC2 is regulated by WNT signaling and it cause SMC2 abnormal expression in CRC cell lines and clinical samples (Tokunaga et al., 2013). These studies suggested that individual human codensin subunit may have a different signal transduction pathway and the same human codensin subunit may be regulated by different signal transduction pathway in colorectal cancer and related disease.

All in one, it opens up a new way for the diagnosis and treatment of colorectal cancer from the point of exploring novel function of human condensin subunits in colorectal cancer. Up to now, it is reported that all subunits of human condensins are involved in the tumorgenensis and can be potential biomarkers and therapeutic targets (Dávalos et al., 2012; Shiheido et al., 2012; Feng et al., 2014; Jinushi et al., 2014; Wang et al., 2018; Baergen et al., 2019; Xiao et al., 2020; Yeh et al., 2020; Zhang et al., 2020). With further research on the molecular mechanism of the eight human condensin subunits in colorectal cancer, more attention will be paid to the development and application of these subunits as potential biomarkers and therapeutic targets in the diagnosis and treatment of colorectal cancer in the foreseeable future.

## Abbreviations

CRC, colorectal cancer; SMC, structure maintenance of chromoses; hCAP, human chromosome associate polypeptide; NCAP, non-SMC condensin I complex subunit; MSI-H, high microsatellite instability; GRID2, glutamate ionotropic receptor delta type subunit 2; CBLN1, cerebellin 1 precursor; POU2F1, TF POU class 2 homeobox 1; HPGDS, hematopoietic prostaglandin D synthase.
